# A Domestic Trash Detection Model Based on Improved YOLOX

**DOI:** 10.3390/s22186974

**Published:** 2022-09-15

**Authors:** Changhong Liu, Ning Xie, Xingxin Yang, Rongdong Chen, Xiangyang Chang, Ray Y. Zhong, Shaohu Peng, Xiaochu Liu

**Affiliations:** 1 School of Mechanical and Electrical Engineering, Guangzhou University, Guangzhou 510006, China; 2 School of Electronics and Communication Engineering, Guangzhou University, Guangzhou 510006, China; 3 School of Environmental Science & Engineering, Guangzhou University, Guangzhou 510006, China; 4 Department of Industrial and Manufacturing Systems Engineering, The University of Hong Kong, Hong Kong 999077, China

**Keywords:** YOLOX, object detection, attention mechanism, deep learning, domestic trash

## Abstract

Domestic trash detection is an essential technology toward achieving a smart city. Due to the complexity and variability of urban trash scenarios, the existing trash detection algorithms suffer from low detection rates and high false positives, as well as the general problem of slow speed in industrial applications. This paper proposes an i-YOLOX model for domestic trash detection based on deep learning algorithms. First, a large number of real-life trash images are collected into a new trash image dataset. Second, the lightweight operator involution is incorporated into the feature extraction structure of the algorithm, which allows the feature extraction layer to establish long-distance feature relationships and adaptively extract channel features. In addition, the ability of the model to distinguish similar trash features is strengthened by adding the convolutional block attention module (CBAM) to the enhanced feature extraction network. Finally, the design of the involution residual head structure in the detection head reduces the gradient disappearance and accelerates the convergence of the model loss values allowing the model to perform better classification and regression of the acquired feature layers. In this study, YOLOX-S is chosen as the baseline for each enhancement experiment. The experimental results show that compared with the baseline algorithm, the mean average precision (mAP) of i-YOLOX is improved by 1.47%, the number of parameters is reduced by 23.3%, and the FPS is improved by 40.4%. In practical applications, this improved model achieves accurate recognition of trash in natural scenes, which further validates the generalization performance of i-YOLOX and provides a reference for future domestic trash detection research.

## 1. Introduction

Along with the development of the economy and the improvement of national quality of life, an extremely large amount of urban trash is inevitably generated in daily life. According to a study, the amount of trash generated worldwide will increase from 2.01 billion tons per year in 2016 to 3.4 billion tons per year in the next 30 years [1]. A large amount of trash pollution will not only cause great harm to people’s physical and mental health but also generate a lot of waste of resources. In order to avoid serious pollution of cities by large amounts of domestic trash, separation of domestic trash is considered to be one of the most effective ways to control environmental pollution [2]. Faced with the problem of trash classification, many countries have formulated various corresponding solutions. For example, in many cities, relevant laws have been promulgated to stipulate that residents need to classify domestic trash manually [3]. In addition, manual sorting is still the main means of trash sorting in most parts of the world, which not only increases the health risk of workers but also increases the related cost compared with automatic sorting by machines. Effective trash management and recycling of waste resources are of great importance for urban development and sustainability. With the technological innovation brought about by mechanical automation and the combination of computer vision and artificial intelligence, automatic trash sorting technology that is more efficient than traditional sorting methods has gradually become more mature. One example is replacing the single-arm trash sorting robot ZenRobotics Recycler and putting it into a variety of complex trash-sorting scenarios for industrial application [4]. Verma et al. [5] proposed a deep learning-based intelligent system for detecting trash with UAV and provided a low-cost, accurate, and easy-to-use solution for effective trash disposal. The combination of deep learning and image processing has greatly facilitated the application of intelligent sorting in new technologies for smart cities.

Object-detection algorithms have appeared long before deep learning. The main task of object detection is to find the location of an object in an image and classify the object. Currently, object detection has become an important research direction in the field of computer vision. The generation of traditional object-detection algorithms is based mainly on image processing and machine learning algorithms. Traditional object-detection algorithms process images mainly by extracting feature points or feature pixel regions using algorithms such as the Harris algorithm, HOG algorithm, and LBP algorithm [6,7,8]. Then, a series of candidate sliding windows are generated by selective traversal algorithm for image detection, and, finally, image regions are classified into candidate frames using algorithms such as support vector machines (SVM) and decision trees [9,10,11]. It can be seen that the object-detection algorithm in traditional image processing is relatively complicated compared with the deep learning object-detection algorithm, which requires manual feature extraction of the image using relevant feature extraction algorithms. In addition, with the object detection in traditional image processing, it is difficult to effectively extract features from images with complex backgrounds, and it is not only difficult to achieve the required accuracy but also has poor generalization ability. With the continuous development of deep learning research, convolutional neural networks are widely used in the fields of object detection and image classification. The convolutional layer in deep convolutional neural networks has a good abstract feature extraction capability, which can represent the high-level semantic information of different layers in a hierarchical feature representation, eliminating the need to extract the image features by other traditional feature extraction algorithms. With the increasing maturity of classification networks such as AlexNet [12], a series of single-stage object detection networks and two-stage object detection networks have followed. The first to appear was the two-stage object-detection network represented by R-CNN [13]. Both Fast-RCNN and Faster-RCNN, improved based on R-CNN, have become object-detection networks represented by high-precision properties [14,15]. The YOLO [16] object-detection network proposed by Redmon et al. in 2016 compensates well for the disadvantage of the two-stage network in terms of detection speed. YOLO as a one-stage detector does not require cascading feature proposers and classifiers but directly classifies and predicts image grid regions, which makes it much faster than two-stage detection networks such as R-CNN. The subsequent SSD series and single-stage detection networks such as YOLOv2 and YOLOv3 have focused on improving the detection accuracy of the network through continuous improvement [17,18,19,20]. Many lightweight network models represented by the MobileNet [21] series draw on the important method of depth wise separable convolution. In addition, Hu et al. [22] proposed the Squeeze-and-Excitation Network in 2017, the first application of attention mechanism in image processing, which improves the expressiveness of the network model and also reduces the parameter redundancy in the network.

With the increasing computing power of GPU and the richness of datasets in recent years, deep learning is widely used in daily life. The application of object-detection models plays a key role in life scenarios. For example, Guo et al. [23] proposed the MSFT-YOLO model for industrial scenarios with large background interference in steel images, easily confused defect categories, large defect scale changes, and poor detection results for small defects. To enhance and preserve the features of small targets, Ye et al. [24] proposed the CAA-YOLO model in combination with high-resolution feature layers to better utilize shallow details and location information. Zhao et al. [25] proposed an improved YOLOv5-based method for accurate detection of wheat spikelet in UAV images, solving the problem of spikelet misdetection and missed detection due to occlusion conditions. Chen et al. [26] achieved real-time detection in surface detection by applying the attention mechanism to the spatial dimension of the model and adding multi-scale feature fusion to the neck structure of the network model. The combination of the above examples of the latest technological methods of mainstream neural networks also allows us to explore the scope for upgrading various neural networks.

Trash detection involves the localization and classification of trash. This application area has been studied by researchers since the beginning of the 21st century, with the increasing awareness of environmental protection in recent years. Earlier work on trash sorting mainly consisted of classifying different single object images and did not include the function of object location detection. In 2016, Yang et al. [27] achieved a test accuracy of 63% by classifying the trash dataset TrashNet with a support vector machine (SVM) coupled with a scale-invariant feature transform (SIFT), a result that surpassed the performance of shallow neural networks in comparison experiments. Later, Zhang et al. [28] constructed a migration learning-based YOLO-WASTE multi-label trash classification model to achieve fast detection and classification of multiple wastes. In 2020, Ye et al. [29] applied Variational Auto-encoder (VAE) to motivate the YOLO network model to learn decomposable representations of complex data, allowing the enhanced model to improve the regression accuracy for trash detection with complex backgrounds. In 2021, to solve the problem of automated separation of degradable and non-biodegradable waste, Karthikeyan et al. [30] used the SSD algorithm and augmented clustering algorithm to achieve automated detection of trash targets. Trash detection faces a number of specific challenges, such as small objects being easily missed, complex image backgrounds, and a lack of good quality datasets. Existing trash-detection models are not yet able to overcome these challenges simultaneously, and there is still much room for improvement in detection accuracy and detection speed.

In this paper, we chose YOLOX, one of the state-of-the-art one-stage detection models available today, as our base model. Although YOLOX is excellent in terms of detection accuracy and detection speed, there is still room for improvement in the application of complex domestic trash scenarios. We proposed i-YOLOX to further improve the detection performance of the base model in complex domestic trash scenarios. Overall, the main contributions we made in this paper include:

(1) We proposed an improved YOLOX-based trash detection model: i-YOLOX. Firstly, we incorporated a new generation of neural network operator involution in the network, which significantly reduced the parameters of the network and improved the accuracy of the model.
(2) The aggregation capability of the deep semantic information of the network was improved by adding the involution CBAM module to feature pyramid networks (FPN) structure. In addition, the capability of classification and regression in decoupled head was improved by adding residual connectivity and involution block.
(3) We also proposed a self-made dataset (CDTD) containing 10,000 samples. We collected a total of 17 common trash images datasets in life scenarios and paid more attention to the collection and labeling of multi-object trash images. In addition, we applied various data enhancement techniques such as Mosaic data enhancement technique to further improve the richness of the dataset.

The remainder of this paper is organized as follows: Related methods about original YOLOX network, involution operator, and CBAM attention mechanisms are introduced in Section 2. Then, the details of improvement in i-YOLOX algorithm are described in Section 3. A detailed description and analysis of the experimental procedure is presented in Section 4. In Section 5, we present extended experiments and discussions. Finally, we summarize our work in Section 6.

## 2. Related Methods

### 2.1. YOLOX Network

The traditional Yolo series network usually first divides the image into a grid of three sizes for distinguishing between large, medium, and small objects. The traditional Yolo series detection network grids the obtained feature layers, and each feature point in the grid corresponds to multiple prior boxes. The Yolo head determines whether the a priori box contains a detection object and then fine-tunes the a priori box for each feature point to obtain the prediction result.

YOLOX [31], as an improved version of Yolo series, surpasses the previous Yolo series networks in several detection metrics. YOLOX is currently one of the best detection network models, and its good detection and recognition results are attributed to the inclusion of several innovative metrics. The feature map is divided efficiently, and then the information of different feature points is stacked onto the channels in order. In addition, the backbone of YOLOX inherits the CSPNet network structure of YOLOv5 [32]. YOLOX embeds the darknet residual block into the CSPNet Bottleneck structure to form a larger residual structure block. Secondly, in the design of the classification and regression layer, unlike the previous Yolo series networks which used a single 1 × 1 convolution for classification and regression, the decoupled head of YOLOX uses two convolutional blocks for classification and regression and finally integrates them together for prediction. In response to the data redundancy caused by the previous Yolo series networks using the anchor-based prior box mechanism, YOLOX uses the anchor-free mechanism not only to simplify the complexity of the Yolo head but also to greatly reduce the time cost. In addition, SimOTA dynamic matching mechanism is one of the important improvements of YOLOX. This method calculates the number of feature points for each ground truth box by judging the overlap between each prediction box and the ground truth box; it then calculates the cost matrix according to the prediction accuracy of each feature point and the inclusion degree of the ground truth box, then the feature points with the lowest cost are used as the positive samples of the ground truth box. Finally, the feature points with the lowest cost are used as positive samples of the ground truth box.

The YOLOX network combines the advantages of the Yolo series networks, using Focus and CSPNet in the backbone for effective feature analysis of the input feature maps and using a decoupled head structure in the detection head. YOLOX with anchor-free and SimOTA mechanisms significantly improves the detection accuracy of the model while reducing the parameter redundancy of the network.

### 2.2. Involution

Involution [33] is a new generation of neural network operators proposed by Li et al. in CVPR2021. It is worth noting that some features of MLP-Mixer [34] are similar to involution. MixLayer in MLP-Mixer is a comprehensive calculation of the information of spatial extent and channel extent, whereas the kernel in involution is more focused on processing the information in the channel dimension. However, in comparing the experiments in the papers of involution and MLP-Mixer, we find that the Avg.5 top-1 performance of MLP Mixer fine-tuned on ImageNet is slightly worse than involution. Thus, we ultimately chose involution for the application in YOLOX. The properties of involution are contrary to traditional convolution. The traditional convolution process follows both spatial-agnostic and channel-specific properties. However, the convolution kernel acts as a fixed filter template and extracts a relatively homogeneous set of features. In addition, the size of the convolution kernel is usually strictly limited due to the number of computational parameters, and multiple 3 × 3 convolution kernels need to be stacked if a large perceptual field is needed. The channel-specific nature suggests that more convolution channels also generate more parameter redundancy.

**Figure 1:**
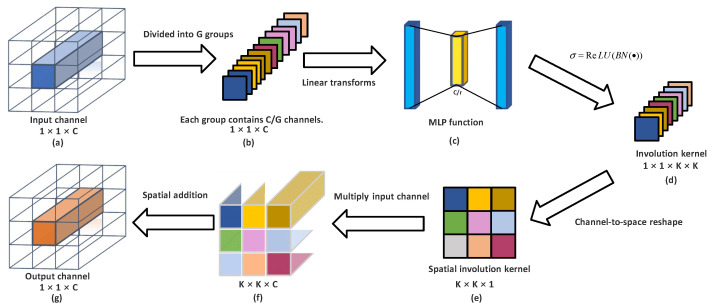
Schematic illustration of involution. (**a**) is the input channel, (**b**) is to divide all input channels into G groups, (**c**) is the linear activation layer, (**d**) is the involution kernel after ReLU activation, (**e**) is the spatial alignment of the involution kernel, (**f**) is the result of multiplying the involution kernel on space with the input channel, and (**g**) is the result of adding (**f**) on the spatial range.

In view of the above limitations of traditional convolution, involution proposed a new research idea: on the premise of ensuring computational efficiency, the long-range and self-adaptive relationship can be better realized in the neural network layer modeling. The design of involution follows two unique properties: spatial-specific and channel-agnostic. The main operation process of involution is shown in the following Figure 1:

First, C channels from the input channel are divided into G groups. The number of blocks in Figure 1b are equal to G, and each block contains C/G channels. Figure 1b shows all channels in Figure 1a are divided equally into multiple groups. The channels within each group share the same kernel, unlike convolution where each channel has a private kernel and the number of groups is much lower than the number of input channels. G is a fixed hyper-parameter, and the smaller the value of G, the more parameters the involution needs to calculate. C/G is the number of kernels of involution. When G is equal to the number of input channels C, the input channels share the same kernel. We set the value of G to 16. Second, the involution kernel is generated by the φ function which is the kernel generation function that makes use of two fully connected layers and obtains the involution kernel by the rectified linear unit (ReLU) activation function. The first linear transformation compresses the channel dimension to C/r, and the second nonlinear transformation makes the vector of C/r as large as K × K × G in the channel dimension. From there we can determine the size of the involution kernel in this point. The parameter r is the compression rate of the linear transformation. The larger the value of the compression rate r, the fewer parameters need to be processed in Figure 1c. The value of r in this work is taken as 4. Considering that the current elements are arranged on channels, we need to rearrange the elements in channel-to-space. Finally, the output feature map of involution is derived by performing multiplication and addition operations on the input channel using involution kernel.

### 2.3. CBAM Attention Mechanism

The attention mechanism is a very effective enhancement structure in deep learning. Usually, the attention mechanism can be divided into two types: spatial attention mechanism and channel attention mechanism. Generally, a feature map block consists of multiple layers of single-channel feature maps stacked together, and the spatial attention mechanism adds weights to the feature points containing object features in a single-channel feature map. The channel attention mechanism makes the feature map block’s high-dimensional semantic features easier to be extracted by the network model by assigning more weights to the feature channels containing feature semantic information.

Briefly, the basic function of the attention mechanism is to make the neural network layer pay more attention to the local features containing the positive features and, thus, improve the representation of the region of interest. When we use convolutional layers to extract image information features, the computational process of feature processing in neural networks becomes more efficient if the network layers can pay attention to effective local features adaptively.

For instance, the SENet [22] proposed by Hu et al. first subjected the feature map to squeezing compression operation, generated channel descriptors by feature aggregation across spatial dimensions, and then selectively emphasized informative features and suppressed irrelevant informative features by learning the activation of specific samples to achieve. The basic mechanism of SENet is to analyze the correlation between different feature channels; Woo et al. proposed CBAM [35] integrating the correlation between feature channels and feature space. Compared with SENet’s attention mechanism which focuses only on feature channels, it can focus on deeper feature semantic information. Wang et al. conducted an in-depth study on the cross-channel interaction process of the attention mechanism and proposed ECA-Net [36] based on the local channel interaction strategy without dimension reduction; this also brought new inspiration to the research of attention mechanism.

**Figure 2:**
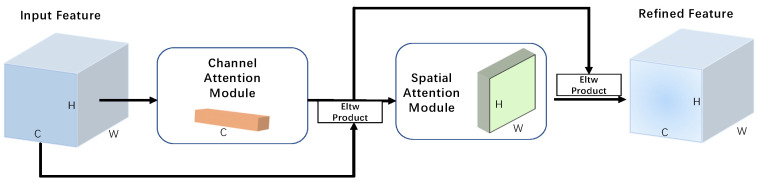
CBAM attention mechanism diagram. The module has two sequential sub-modules: channel attention module and spatial attention module. H is height, W is width, and C is channel. Eltw is element-wise.

Since this paper takes into account too heavily the surrounding context information after introducing involution CSPLayer, it causes the interference of local information. To handle this, we applied the CBAM (as shown in Figure 2) into our model which can effectively deal with such a problem. Through our experiments, we found that CBAM works better than other attention mechanisms in our model, and the detection accuracy of objects is also improved.

## 3. i-YOLOX

### 3.1. Overview of i-YOLOX

**Figure 3:**
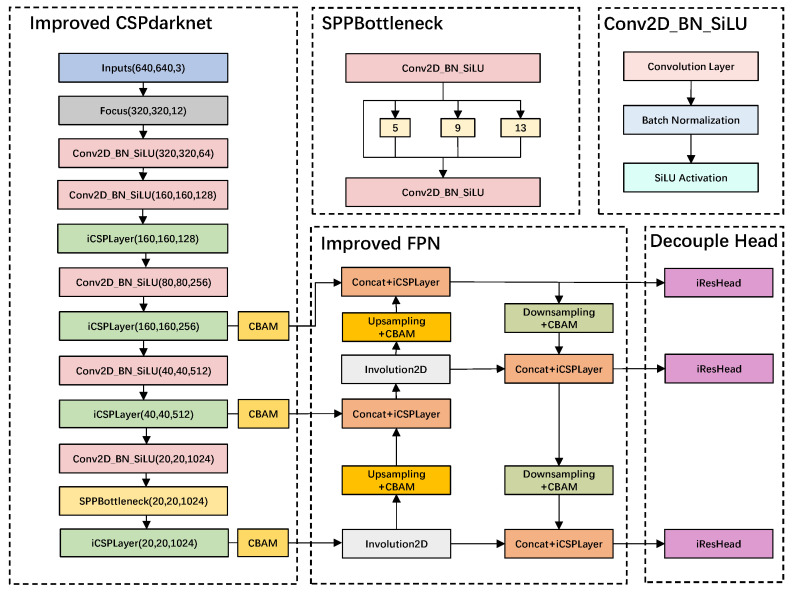
i-YOLOX network architecture diagram. Improved CSPdarknet is the backbone of i-YOLOX. FPN is feature pyramid network. Decoupled head is the detection of i-YOLOX. SPP is spatial pyramid pooling. Conv2D_BN_SiLU is a stacked structure containing a two-dimensional convolutional layer, a batch normalization layer, and a sigmoid-weighted liner unit layer.

i-YOLOX is an improved detection model based on YOLOX. In our experiments, the detection result of i-YOLOX is better than the original YOLOX detection model. We optimize the CSPLayer structure in the backbone of YOLOX by incorporating the involution operator in the residual module of the CSPLayer structure. Second, three CBAM attention mechanism modules are added to the outputs of the backbone to further improve the efficiency of feature extraction in the backbone. In addition, the involution CBAM block is incorporated to the PANet structure to strengthen the feature extraction of the network by adding the attention mechanism. Finally, an involution residual decoupled head is proposed, which further improves the performance of i-YOLOX. The structure of i-YOLOX is shown in Figure 3:

### 3.2. Improved CSPLayer

**Figure 4:**
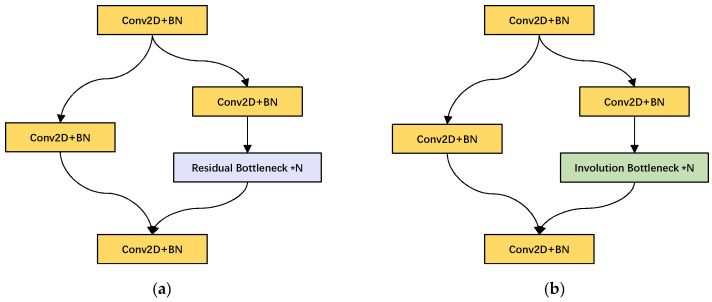
CSPLayer structure diagram. (**a**) is the original CSPLayer and (**b**) is the improved CSPLayer.

The backbone of YOLOX is mainly composed of the composite residual structure of CSPLayer. CSPLayer is basically divided into two parts; the specific structure is shown in Figure 4a. The backbone part consists of a shallow convolutional layer and a sub-residual block. The residual part is simply processed by a 1 × 1 convolution layer and directly connected to the CSPLayer output part. In i-YOLOX, we replace the residual bottleneck module in CSPLayer with the involution bottleneck module (as shown in Figure 4b). In addition, in order to strengthen the feature extraction layer later to better pay attention to the important feature parts of the three involution CSPLayer (iCSPLayer) output feature layers, we also append CBAM attention mechanism modules after the three iCSPLayer of the backbone, respectively.

**Figure 5:**
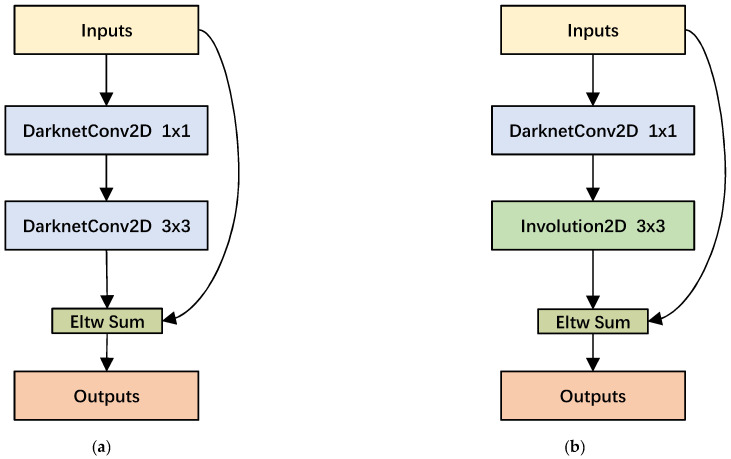
Sub-residual block structure diagram. (**a**) original bottleneck. (**b**) improved bottleneck.

The sub-residual bottleneck structure is an important part of the backbone feature extraction network, and the structure is shown in Figure 5a. Most of the feature extraction process of the network is carried out in this structure. The stacked layers of the residual bottleneck consist of a 1 × 1 convolutional layer and a 3 × 3 convolutional layer. Additionally, shortcut connections are applied to straightly add elements to the output of the convolutional layer. The application of the residual structure greatly alleviates the gradient disappearance problem caused by the deepening process of the neural network.

The 3 × 3 convolutional layer in the residual structure needs to handle a large number of parameter operations, which also causes a great deal of parameter redundancy. Therefore, we introduce an involution operator with adaptive relational modeling, which can greatly reduce the parameter amount of bottleneck. Furthermore, involution has the capability of sharing semantic information across channels, which is beneficial to extract object features in the actual complex trash background. We replace the 3 × 3 DarknetConv2D in the sub-residual bottleneck with the Involution2D layer, and the experimental results showed the efficiency brought by involution. The involution sub-residual bottleneck structure is shown in Figure 5b:

### 3.3. Improved Feature Pyramid Network

Feature pyramid network (FPN) [37] is the feature enhancement extraction module of YOLOX, which enhances feature extraction for the three CSPLayer output feature layers of the backbone, respectively. After obtaining the three effective feature layers of the backbone, the three effective feature layers are used to construct the FPN layer. The FPN in this paper uses the PANet [38] structure. The purpose of designing this structure is to enable the decoupled head to further extract more obvious and effective features from the rich semantic information and perform effective feature fusion. The input feature layers of PANet come from the middle layer, the lower layer, and the bottom layer of the backbone, respectively. PANet first fuses the deep features of the backbone with the shallower features through the up-sampling module in turn, and then aggregates the low-level feature layers through the downsampling module. Such a combination of up-sampling and down-sampling operations helps the network to extract better features.

**Figure 6:**
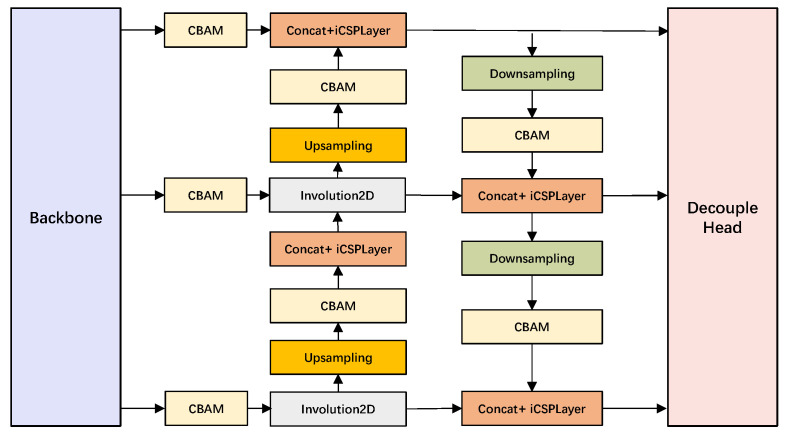
iPANet structure diagram. CBAM is convolutional block attention module. Involution2D is two-dimensional involution layer. Concat means stacking channels together.

Since we use the involution CSPLayer structure in the backbone network, the convolutional layer of the backbone contains a large amount of complicated semantic information. At this time, if the feature enhancement extraction is performed directly on the backbone output layer, the feature extraction efficiency will become lower. We deeply incorporate the convolution block attention module (CBAM) attention mechanism in our improved PANet. The purpose is to make the enhanced feature extraction network adaptively focus on the important feature parts in the feature layer. We incorporate four CBAM attention modules in PANet. The four CBAM modules are arranged after the up-sampling module and the down-sampling module, respectively. Although multiple CBAM modules are added, our experimental results show that the CBAM module adds only a very small number of parameters to the network. In addition, we replace the Conv2D module in PANet with the Involution2D module, which also reduces the number of parameters for a large number of enhanced feature extraction networks. The structure of improved PANet (iPANet) is shown in Figure 6:

### 3.4. Involution Residual Decoupled Head

The conflict between classification tasks and regression tasks is a very typical problem in the field of object detection [39]. In most one-stage and two-stage detectors today, decoupled heads are usually used for classification and regression. The structure of the coupled detection head of the traditional YOLO series network would restrict the performance of the network, and the detection head structure design of the decoupled head is used in YOLOX.

**Figure 7:**
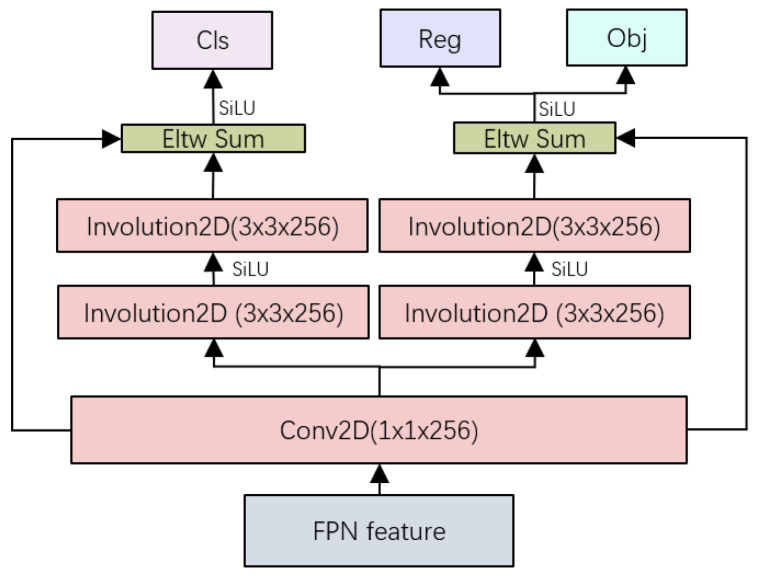
iResHead structure diagram. Cls is classification. Reg is regression. Obj is objection.

Inspired by the design of the prediction module in DSSD [20], we designed the involution residual decouple head (iResHead) in i-YOLOX (as shown in the Figure 7). Conv2D (1 × 1 × 256) was used to reduce the number of FPN feature channels to 256. We added a shortcut connection between the Conv2D input layer and the Involution2D output layer, which reduced the occurrence of gradient disappearance during backpropagation. We used element-wise sum (Eltw Sum) at the Involution2D output location for the connection. At the same time, we replaced Conv2D (3 × 3 × 256) in the decoupled head with Involution2D (3 × 3 × 256). Our experimental results showed that the improved decoupled head not only maintained the accuracy of the original structure but also significantly reduced the number of parameters.

## 4. Experimental Procedure and Results

### 4.1. Datasets and Evaluation Criteria

In this section, we perform detailed experiments and analysis of the results. Detailed information about the trash detection dataset we produced and the evaluation criteria of the object detection are presented in Section 4.1. Then, we describe the experimental procedure and perform the analysis of the experimental results in Section 4.2. There are four subsections in Section 4.2. In Section 4.2.1, we present our experimental equipment parameters and training settings. In Section 4.2.2, we compare the performance of our i-YOLOX model with other state-of-the-art detection models. In Section 4.2.3, we perform ablation experiments based on our method. In Section 4.2.4, we perform a comparative analysis of the performance of different models for practical applications.

#### 4.1.1. Dataset

**Figure 8:**
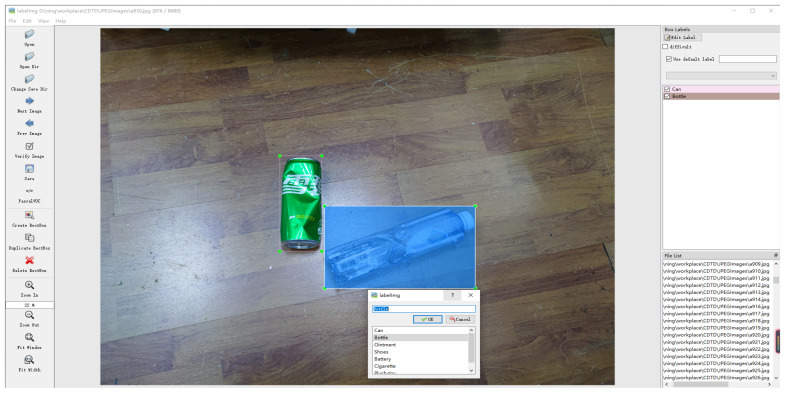
LabelImg software operating interface.

In the work related to deep learning, datasets play a crucial role. Richer datasets mean that the trained model can learn more features. In our work, a common domestic trash dataset (CDTD) covering 17 domestic trash types is presented, which contains a total of 10,000 detection objects. These 17 common domestic trashes are: shoes, cutting board, can, bottle, battery, clothes, cigarette, ointment, wash supplies, plastic toy, courier bag, power bank, pillow, pot, plastic hanger, tea residue, and plush toy. These dataset images were obtained mainly by taking pictures with our camera and collecting them from the internet. We label the obtained images with LabelImg software and save the object information as XML files (as shown in Figure 8, the Chinese on the green can is Sprite.). In addition, we randomly use 10% of the obtained image dataset for the test dataset, then 10% of the remaining images for the validation dataset, and all the remaining images for the training dataset. The training dataset will be used to train the neural network to obtain the weight parameters. The validation dataset is used to test the training process and adjust the training parameters. The test dataset is prepared for the final test of the generalization performance of the trained model.

Before using the dataset for training, our dataset undergoes a series of preprocessing. This preprocessing can largely reduce the invalid image information, which not only can reduce the parameter computation during model training but also can improve the generalization performance of the training model. In addition, we employ various data augmentation methods to enhance the diversity of the dataset. These data augmentation techniques mainly include cropping, rotating, flipping, scaling, and mosaic splicing [40], which are effective in reducing the occurrence of overfitting of the training data. The specific preprocessing steps are shown below:

(1) Convert the input RGB image to a single channel grayscale image and filter the grayscale image using Gaussian blur.
(2) Randomly change the height and width of the input image so that the side length of a single image can be changed to multiple proportions.
(3) Rotate the input image to several different angles.
(4) Use the mosaic stitching technique to randomly stitch four images into a new image.
(5) Trim the processed image to a 640 × 640 RGB image. If the length or width of the processed image is less than 640, change the image size to 640 × 640 by filling with gray bars.

#### 4.1.2. Evaluation Criteria

The performance of object detection usually needs to be evaluated with certain criteria; therefore, some standard evaluation indicators for object detection are introduced next.

**Figure 9:**
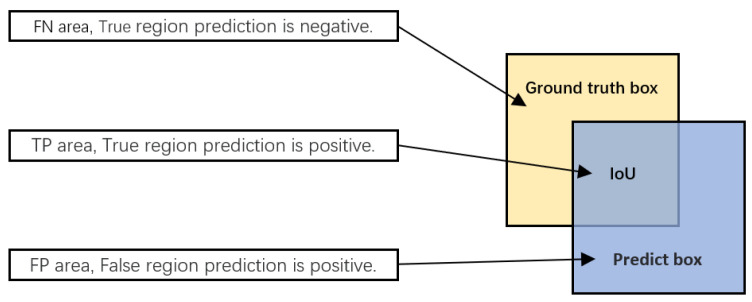
IoU stands for Intersection over Union. Ground truth box denotes the true label of the dataset object. Predict box denotes the result of the model predicting the object.

(1) We first need to know how to indicate the difference between the prediction and the true value, and we define *FN*, *TP*, and *FP* of Figure 9: *F* and *T* stand for False and True, and *N* and *P* mean negative and positive, respectively. *F* indicates that the prediction result does not match the true value, and *T* indicates that the prediction result is consistent with the true value. Positive means the prediction result is positive category, and Negative means the prediction result is negative category.

(2) Precision (*P*) is used to measure how many of the predicted positive classes are true positive classes (as shown in Equation (1)). Recall (*R*) is used to measure the proportion of true positive classes being recalled (as shown in Equation (2)). Precision and Recall sometimes contradict each other, and they need to be considered together. *F1* is used to comprehensively evaluate the results of Precision and Recall (as shown in Equation (3)). When *F1* is higher, it means that the experimental model has higher accuracy.

(1) $$P=\frac{TP}{\left(TP+FP\right)}$$

(2) $$R=\frac{TP}{\left(TP+FN\right)}$$

(3) $$F1=\frac{2PR}{\left(P+R\right)}$$

(3) Average Precision (*AP*) is the average precision over all Recall values between 0 and 1 (as shown in Equation (4)). *AP* can intuitively reflect the performance of the object detector. Mean Average Precision (*mAP*) represents the mean of *AP* for all classes in the evaluated dataset (as shown in Equation (5)). In Equation (5), *N* represents the categories of objects in the dataset.

(4) $$AP=\int_{0}^{1}P(R)dR$$

(5) $$mAP=\frac{\sum_{i=1}^{N}AP_{i}}{N}$$

### 4.2. Experimental Analysis

#### 4.2.1. Training Parameters

Our research used the Pytorch deep learning framework to design and implement our algorithm. The device parameters used to train our model are specified in Table 1 below. We chose to use stochastic gradient descent to optimize the parameters during training [41]; we set the momentum to 0.93 and the weight decay parameter to 0.0005. In addition, we used a dynamic learning rate for training, the learning rate was initialized to 0.01, and then the learning rate was reduced by applying a cosine learning rate strategy [42]. The batch size was set to 36, and the number of workers was set to 30 to load the data using multiple threads. All models were trained for a total of 150 epochs, where one epoch means that the entire training dataset is input to the network for training once.

#### 4.2.2. Ablation Experiments

In this section, in order to better analyze the impact of each proposed scheme on the model, the ablation experiments were carried out based on the self-built domestic trash dataset. Our modification strategy is shown in Table 2. Then, we compared the resultant parameters of different schemes for different YOLOX-based improvement models in Table 3. These specialized parameters included precision (P), recall (R), F1, mean average precision (mAP), parameters, giga floating point operations per second (GFLOPs), and frames per second (FPS). These parameters allowed us to clearly see the enhancement effect of each enhancement scheme on the original YOLOX model.

Specifically, in the scheme iCSPLayer, we replaced the 3 × 3 convolution layer in the bottleneck of the original CSPLayer with the 3 × 3 involution layer, and the other 1 × 1 convolution layer remained unchanged. In addition, we added CBAM modules to the three iCSPLayer output positions of the backbone. In scheme iPANet, a modified PANet was applied to the feature pyramid network (FPN) structure. First, the CBAM module was added after each up-sampling module and down-sampling module of iPANet. Then, the 3 × 3 convolution layer in the original PANet was replaced with a 3 × 3 involution layer. In the scheme residual head, we used shortcut connection after the 1 × 1 convolution layer in the decoupled head to connect to the last 3 × 3 convolution layer for element-wise sum with the output feature map. In the final involution head scheme, we replaced the feature classification convolution layer in the middle part of the decoupled head with a lightweight involution layer. The combination of the above four methods is our proposed improved algorithm (named i-YOLOX).

The results of the ablation experiments are shown in Table 3, where we can see that the original YOLOX algorithm had low values of the included P, R, F1, and mAP values. In addition, there was space for reducing the number of parameters and GFLOPs of the original YOLOX. Compared with the original algorithm, the enhanced iCSPLayer-based YOLOX algorithm made the network significantly lighter because of the channel reduction of the evolution operator in the iCSPLayer, which reduced the number of parameters and GFLOPs of the network. Benefiting from two special properties of involution, spatial-specific and channel-agnostic, iCSPLayer can combine complex semantic information among different channels of trash images, which also allowed our model to analyze the object information of more complex scenes in images. Scheme iCSPLayer increased P by 0.65%, F1 by 1.36%, and mAP by 0.47%, while the number of parameters decreased by 1.82 million. It is worth noting that the FPS of scheme iCSPLayer was improved by 17.13 with respect to baseline. Scheme iCSPLayer-iPANet enhanced the feature extraction part of the FPN structure by adding a CBAM attention module that combined both spatial and channel dimensions to focus on the effective features; this allowed the FPN structure to better integrate shallow feature information with deep feature information. Although the seven CBAM modules added to iPANet increased the number of network parameters by 1.3 million, iPANet resulted in a 0.14% improvement in mAP. Based on scheme iCSPLayer-iPANet, shortcut connection was introduced into the decoupled head structure, and ResHead increased mAP by 0.7% and improved FPS by 6.87% compared with scheme iCSPLayer-iPANet with the same number of parameters. Finally, compared with scheme iCSPLayer-iPANet-ResHead, scheme iCSPLayer-iPANet-iResHead replaced the 3 × 3 convolution layer in the decoupled head with an involution layer, which improved P, F1, and mAP by 1.29%, respectively. In addition, mAP increased by 1.29%, 0.73%, and 0.16%, respectively, while the number of parameters was reduced by 1.56 million. Although the FPS of scheme iCSPLayer-iPANet-iResHead decreased by 5.32 compared to scheme iCSPLayer-iPANet-ResHead, it still improved by 10.64 compared to baseline. Thanks to the combination of the above proposed methods, i-YOLOX showed significant advantages in terms of algorithm accuracy and model complexity compared with the original YOLOX algorithm. i-YOLOX showed significant advantages in terms of algorithmic accuracy and model complexity. Overall, the performance of our model in terms of validation dataset increased by 1.94% for P, 1.9% for R, 2.09% for F1, 1.47% for mAP, and 10.64 for FPS, while the number of parameters and GFLOPs decreased by 2.08 million and 9.51, respectively.

**Figure 10:**
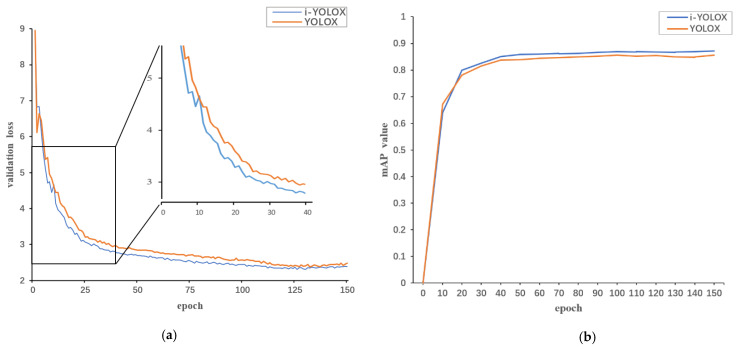
(**a**) is validation loss curve plot, (**b**) is mAP curve plot.

To further describe the enhancement effect of our method on the original YOLOX model, we also compared the training loss function value plot (as shown in Figure 10a) and the mAP varying plot (as shown in Figure 10b) of i-YOLOX with the original YOLOX model. In Figure 10a, we can see the curves of the training loss value and validation loss value with epoch for the original YOLOX and i-YOLOX. The convergence of the loss values can objectively reflect how effectively the deep learning algorithm learns the features, where usually the faster the loss curve converges, the more efficient the algorithm is in learning. We can see that both i-YOLOX validation loss value curves converge faster than the original YOLOX curves. Therefore, we can conclude that the training effect of the i-YOLOX model was better than the original YOLOX model. In Figure 10b, we compare the mAP values with epoch for i-YOLOX and the original YOLOX. mAP is a comprehensive way to evaluate the model precision and recall; the mAP value is also one of the most important indicators of the performance of an object-detection model. From Figure 10b, we can see that the change curve of mAP value of i-YOLOX is eventually higher than the mAP value of the original YOLOX, which indicates that i-YOLOX can achieve better detection accuracy requirements. From the change of the loss function value and the change of the mAP value, it can be seen visually that the final improved algorithm model was better than the original algorithm model.

In this experiment, all the compared algorithms were trained for 150 epochs. The training time was the time it took to train the algorithm for 150 epochs. In addition, the test times of the original and improved algorithms were also calculated. The test time was calculated as the average time taken by the algorithm to detect 100 images from the test set. The specific experimental time parameters are shown in Table 4:

#### 4.2.3. Result Comparison with Other Detection Algorithms

To better demonstrate the superiority of our algorithm, we also compared and analyzed the combined performance of different popular object-detection algorithms on top of the test dataset. As shown in Table 5, we compared the different algorithm models in terms of precision (P), recall (R), F1, mean average precision (mAP), parameters, giga floating point operations per second (GFLOPs), and frames. The experimental results of the different algorithms are listed in the table in decreasing order of the number of parameters. From the experimental results in Table 5, we were able to perform the following analysis. First, Faster-RCNN, as one of the representatives of two-stage detectors, had a much lower number of parameters and GFLOPs than other one-stage detectors, although its mAP reached 81.70%. The number of parameters of Faster-RCNN was 19.98 times higher than that of our algorithm. Second, we found that although YOLOv4 and YOLOv5 were much faster than Faster-RCNN, their mAP values were lower than Faster-RCNN. In the SSD comparison experiment, we found an interesting observation: although the number of parameters and GFLOPs of SSD were higher than those of YOLOX and i-YOLOX, the FPS of SSD was higher than those of YOLOX and i-YOLOX. Our analysis suggests that the reason for this phenomenon is that there was no FPN feature enhancement extraction process in the structure of SSD, so the detection speed of SSD was much faster. Although YOLOX showed high performance in all metrics, i-YOLOX algorithm still showed many improvements compared with the original YOLOX algorithm. In summary, i-YOLOX outperformed the other exemplified algorithms on the CDTD dataset in aggregate.

#### 4.2.4. Detection Results under Application Scenarios

**Figure 11:**
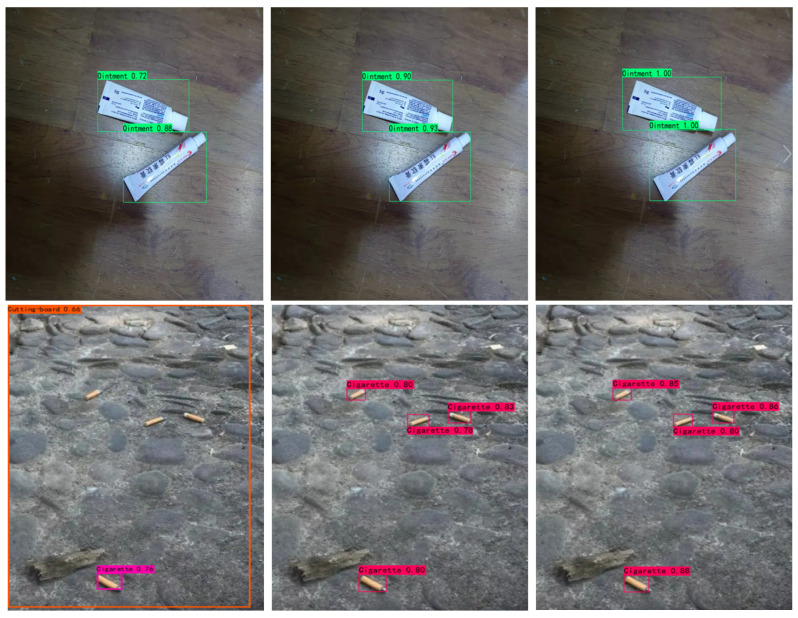

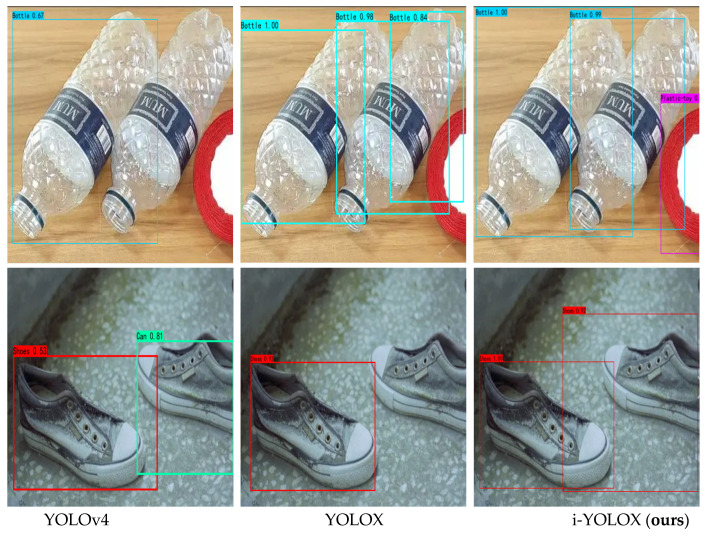
The detection results of test domestic trash images.

To display the advantages of our enhanced algorithms in terms of detection performance in real-life scenarios, we applied different object-detection algorithms to perform object detection on some of the test images. As can be seen in Figure 11, we used three algorithms, YOLOv4, YOLOX, and i-YOLOX, to recognize four groups of common domestic trash images. In the first set of images, although all algorithms correctly identified all the ointment objects, i-YOLOX performed better than the other two algorithms in terms of accuracy. In the second set of images, YOLOv4 detected only one cigarette butt and there was a false detection identified as a cutting board. While both YOLOX and i-YOLOX identified all cigarette butts, i-YOLOX was still more accurate than YOLOX. From the results of the second set of comparison tests, we found that i-YOLOX showed superior performance to the original YOLOX in the detection of small objects. In the third set of images, YOLOv4 recognized only one bottle, and YOLOX falsely detected the plastic toy as a bottle. In the fourth set of images, YOLOv4 identified only one shoe correctly, but the other shoe was mistakenly detected as a can. YOLOX missed one shoe detection, whereas i-YOLOX identified two shoes. From these four sets of detection comparison experiments we can clearly see that i-YOLOX was more accurate than the original YOLOX algorithm and outperformed the other algorithms we have demonstrated.

## 5. Discussion

In our work, there are two main research lines. The first research direction is in the direction of model lightweighting. We work on detection algorithms that can be more easily deployed into vision sensing device applications. Therefore, we chose to start our research using the lightweight features of methods such as YOLOX and the involution mechanism. The second research direction is to improve the generalization ability of the model. Since we started our research from the perspective of trash detection applications, we have become more interested in algorithms to identify various kinds of trash with large intra-class differences. Additionally, the spatial-specific and channel-agnostic properties of involution is beneficial for the algorithm to perform long-range and self-adaptive feature analysis. We combine the above two main ideas for algorithm performance improvement. We can also see from the experimental results based on CDTD that i-YOLOX is indeed suitable for trash detection. To further discuss the generalization performance of i-YOLOX, our network will be subjected to comparative experiments on TrashNet.

TrashNet [27] is one of the most classic public datasets in the field of trash detection and classification. The TrashNet dataset contains six types of trash images: glass, metal, plastic, paper, cardboard, and trash. As one of the most classic datasets, many researchers use TrashNet to test the performance of the network. We selected some of the state-of-the-art algorithms for comparison so as to exemplify the generalization performance of i-YOLOX. Because all the images contained in the TrashNet dataset are single object images, we compared the object detection algorithm and the classification algorithm together. Table 6 shows the experimental results on the TrashNet dataset:

All detectors and classifiers in Table 6 are experimental results based on the TrashNet dataset tests. The value names in the second column of the table are Accuracy/mAP because the accuracy measures of the detectors and classifiers are different. Usually, we use accuracy to measure the accuracies of classifiers and mAP to measure the accuracies of detectors. We can use these two metrics to visualize the high and low performance of different CNN algorithms. In addition, we did not use metrics such as Precision, Recall, or FPS for comparison because of the different measures of detectors and classifiers. In the table, because each CNN algorithm is mainly influenced by training times and optimizers during training, we identified them accordingly. As we can see from the experimental results in the table, our algorithm achieved relatively good performance on TrashNet for both detectors and classifiers.

**Figure 12:**
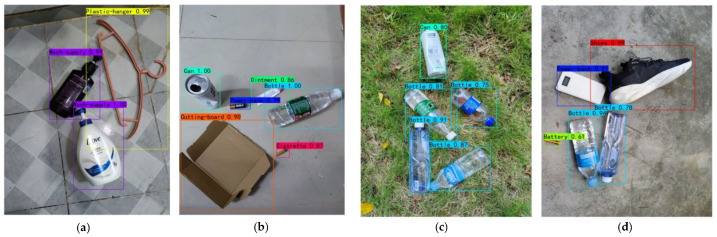
Application of i-YOLOX in multi-object scenarios. (**a**) is a bathroom scene to detect wash-supply and plastic-hanger. (**b**) is a corridor scene to detect cutting-board, can, ointment, bottle, cigarette and battery. (**c**) is the detection of can and bottle in the grass scene. (**d**) is a concrete floor scene to detect power-bank, shoes, battery and bottle.

Further, the dataset played an important role in the experiment. The effect of the dataset on the experiment is also a topic worth discussing. The rich dataset facilitated the construction of more robust detection models. Datasets such as TrashNet are still far from meeting the needs of domestic trash detection. The small number of images in TrashNet and the fact that they are all single object images led to poor generalization of the training model. In other words, we need larger, more labeled, and more diverse datasets. The CDTD dataset proposed in this paper makes up for the lack of trash detection datasets at the present time. In real scene detection, the CDTD dataset enabled our algorithm to generalize which allowed us to detect more objects more accurately. The detection results of i-YOLOX in a multi-object detection scenario are shown in Figure 12. We can see that the overall detection result was effective. In addition, we notice that false detections inevitably occur in practical applications. For example, the battery in Figure 12b was mistakenly detected as a power bank. Therefore, we should consider enhancing the collection of small object samples in our further work, thus making the model more capable of detecting small targets. Although CDTD dataset contains 17 labels and 10,000 sample objects, more research is required to collect a richer dataset of trash images in the future.

## 6. Conclusions

In this paper, to improve the performance of the object detection algorithm for urban domestic trash object detection, we incorporated various advanced techniques based on the YOLOX-S algorithm, such as the lightweight involution CSPLayer structure (iCSPLayer), the efficient convolution block attention module (CBAM), and the decoupled head structure with shortcut connection (iResHead). We carried out several ablation experiments and results analyses on different object detection algorithms using a self-made domestic trash dataset, CDTD. We found that i-YOLOX achieved better detection performance in terms of model lightweighting and accuracy than other object detection algorithms. In addition, we incorporated the involution operator into the i-YOLOX algorithm thus enabling the model to reduce a large number of parameters while significantly improving the detection speed of the model. The lightweight of the algorithm enabled the implementation of remote sensing devices as well as the implementation of devices such as unmanned robots, which provided algorithmic support for the development of smart city technology. Our study provides some reference value for future research on model lightweighting of detection algorithms. In the next work, we will continue to enrich our dataset of domestic trash images and further simplify the complexity of the network structure to improve the detection speed of the algorithm.

## Figures and Tables

**Table 1:** Experimental equipment and environmental settings.

Parameter	Configuration
CPU	Intel Xeon Gold 6248R
GPU	NVIDIA RTX A6000 (48 GB)
CUDA version	CUDA 11.1
Python version	Python 3.8
Deep learning framework	Pytorch 1.8.1
Operating system	Ubuntu 20.04.3 LTS

**Table 2:** Different schemes based on YOLOX.

Scheme	iCSPLayer	iPANet	Residual Head	Involution Head
iCSPLayer	√			
iCSPLayer-iPANet	√	√		
iCSPLayer-iPANet-ResHead		√	√	
iCSPLayer-iPANet-iResHead	√	√	√	√

**Table 3:** Results of ablation experiments. The bolded parameters are the most optimal.

Method	P/%	R/%	F1/%	mAP/%	Parameters/M	GFLOPs	FPS
YOLOX(baseline)	85.78	80.06	82.58	85.68	8.94	26.79	26.37
iCSPLayer	86.43	81.92	83.94	86.15	7.12	21.86	**43.50**
iCSPLayer-iPANet	87.47	79.34	83.09	86.29	8.42	25.97	35.46
iCSPLayer-iPANet-ResHead	86.43	81.92	83.94	86.99	8.42	25.97	42.33
iCSPLayer-iPANet-iResHead	**87.72**	**81.96**	**84.67**	**87.15**	**6.86**	**17.28**	37.01

**Table 4:** Training time and testing time.

Methods	Training Time	Testing Time
YOLOX	26.00 h	0.0379 s
i-YOLOX	13.25 h	0.0270 s

**Table 5:** Comparison with other algorithms. The bolded parameters are the most optimal.

Algorithm	P/%	R/%	F1/%	mAP/%	Parameters/M	GFLOPs	FPS
Faster-RCNN [14]	54.67	**86.14**	66.67	81.70	137.04	370.14	11.79
YOLOv4 [40]	84.34	58.68	67.74	75.13	64.02	60.07	16.98
SSD [17]	84.15	77.15	80.25	82.08	25.88	62.45	**40.13**
YOLOv5 [32]	83.30	54.49	64.47	75.63	47.06	115.91	24.55
YOLOX [31]	85.78	80.06	82.58	85.68	8.94	26.79	26.37
i-YOLOX (**ours**)	**87.72**	81.96	**84.67**	**87.15**	**6.86**	**17.28**	37.01

**Table 6:** Comparison with other detection and classification algorithms.

Algorithm	Accuracy/mAP	Training Times	Optimizer	Type	Model Size
i-YOLOX (**ours**)	97.57	100 epochs	Adam	detector	26.5 MB
YOLOX [31]	97.36	100 epochs	Adam	detector	34.3 MB
Faster R-CNN [14]	95.83	100 epochs	Adam	detector	108 MB
SSD [17]	93.34	100 epochs	SGD	detector	98.7 MB
YOLOv5 [32]	88.52	100 epochs	SGD	detector	27.3 MB
YOLOv3 [43]	81.36	12,000 iterations	SGD	detector	235 MB
ResNet50 [44]	91.40	40 epochs	Adadelta	classifier	97.2 MB
RecycleNet [45]	81.00	200 epochs	Adam	classifier	-
DenseNet121 [45]	95.00	200 epochs	Adam	classifier	30.8 MB
Inception V4 [46]	94.00	120 epochs	Adam	classifier	174 MB
VGG19 + SoftMax [47]	87.90	100 epochs	Adam	classifier	-

## Data Availability

The data that support the findings of this study are available from the corresponding author upon reasonable request.
